# Metals of Deep Ocean Water Increase the Anti-Adipogenesis Effect of *Monascus*-Fermented Product via Modulating the Monascin and Ankaflavin Production

**DOI:** 10.3390/md14060106

**Published:** 2016-05-27

**Authors:** Tzu-Ying Lung, Li-Ya Liao, Jyh-Jye Wang, Bai-Luh Wei, Ping-Yi Huang, Chun-Lin Lee

**Affiliations:** 1 Department of Life Science, National Taitung University, 369, Section 2, University Rd., Taitung 95092, Taiwan; luck6950@gmail.com (T.-Y.L.); bigli10118@livemail.tw (L.-Y.L.); blwei@nttu.edu.tw (B.-L.W.); 2 Department of Nutrition and Health Science, Fooyin University, Kaohsiung 83102, Taiwan; FT054@fy.edu.tw; 3 Water Resource Division, Stone and Resource Industry R&D Center, Hualian 973, Taiwan; koh@srdc.org.tw

**Keywords:** deep ocean water, obesity, *Monascus*, monascin, ankaflavin

## Abstract

Deep ocean water (DOW) obtained from a depth of more than 200 m includes abundant nutrients and minerals. DOW was proven to positively increase monascin (MS) and ankaflavin (AK) production and the anti-adipogenesis effect of *Monascus*-fermented red mold dioscorea (RMD). However, the influences that the major metals in DOW have on *Monascus* secondary metabolite biosynthesis and anti-adipogenesis remain unknown. Therefore, the major metals in DOW were used as the culture water to produce RMD. The secondary metabolites production and anti-adipogenesis effect of RMD cultured with various individual metal waters were investigated. In the results, the addition of water with Mg, Ca, Zn, and Fe increased MS and AK production and inhibited mycotoxin citrinin (CT). However, the positive influence may be contributed to the regulation of pigment biosynthesis. Furthermore, in the results of cell testing, higher lipogenesis inhibition was seen in the treatments of various ethanol extracts of RMD cultured with water containing Mg, K, Zn, and Fe than in those of RMD cultured with ultra-pure water. In conclusion, various individual metals resulted in different effects on MS and AK productions as well as the anti-adipogenesis effect of RMD, but the specific metals contained in DOW may cause synergistic or comprehensive effects that increase the significantly positive influence.

## 1. Introduction

Deep ocean water (DOW) generally means ocean water from a depth of more than 200 m. The character of DOW includes abundant nutrients and minerals [1,2]. Currently, DOW has been applied to the food, agriculture, cosmetic, and medical fields due to its high contents of minerals such as magnesium (Mg), calcium (Ca), potassium (K), zinc (Zn), *etc.* [3,4,5,6,7,8,9,10,11]. The applications of DOW on fermentation study are currently few. However, these rich minerals in DOW are able to promote the growth rate or metabolite production of microorganisms via acting as the co-factors of the key enzymes [12,13,14,15]. Based on these reasons, the application of DOW to the stimulation of biomass formation and functional metabolites production in microorganisms is probably useful.

*Monascus* species have been used as a traditional food fungus in Eastern Asia for several centuries. *Monascus*-fermented products have gradually developed as a popular and important functional food for the prevention of cardiovascular disease [16]. Red mold dioscorea (RMD) were proven as the strong hypolipidemic functional food in a previous study [17]. However, we found that RMD had a weak effect on anti-obesity, which limited the development of RMD for the prevention of metabolic syndrome. Monascin (MS) and ankaflavin (AK) isolated from *Monascus*-fermented product were proven to prevent obesity development via the suppression of differentiation and lipogenesis in our *in vitro* and *in vivo* studies [18,19]. Therefore, enhancing MS and AK levels in RMD may enhance the anti-obesity effect of RMD.

Previous studies have verified that DOW can facilitate *Monascus*-fermented RMD to biosynthesize high quantities of MS and AK, and low-mycotoxin citrinin (CT) level [13,20]. Wang *et al.* (2013) revealed that ROW-RMD did not significantly reduce weight gain, fat pads, or the size and number of adipocytes, whereas a significant reduction was achieved by the application of RMD cultured in DOW (DOW-RMD) [21]. *In vitro* tests further showed that this effect can be explained by the superior suppression in the preadipocytes’ differentiation function and the lipoprotein lipase (LPL) activities of mature adipocytes. However, the superior anti-obesity effect of DOW-RMD is likely because of the high quantities of MS and AK in DOW-RMD [21]. Numerous studies have also indicated that the metals in DOW may be one of the primary factors influencing organism growth and compound generation [2,13,14]. However, the influences that the major metals in DOW have on *Monascus* secondary metabolite biosynthesis and various health functions remain unknown. In addition, whether major metals influence the anti-obesity effect of RMD remains unclear. Therefore, this study cultivated *Monascus purpureus* NTU 568 by using ultrapure water (UPW), DOW, the various individual metals in water (including Mg, Ca, Na, K, Zn, or Fe contained in DOW), and synthetic water (SW) including a mixture of the six metals in preparation for the fabrication of RMD. This study examined the influence that DOW, SW, and various metals in water had on the biosynthesis of secondary metabolites (*i.e.*, MS, AK, and CT), as well as the various influences that the RMD ethanol extract (RE) samples that were cultivated by using these metals had on inhibiting the differentiation and adipogenesis of 3T3-L1 preadipocytes. In this context, the primary objective of this study was to determine the roles that the various major metals contained in DOW play in the metabolite biosynthesis and anti-obesity effect of DOW.

## 2. Results

### 2.1. Effect of DOW on the Biosynthesis of Monascus-Fermented Metabolites

**Figure 1:**
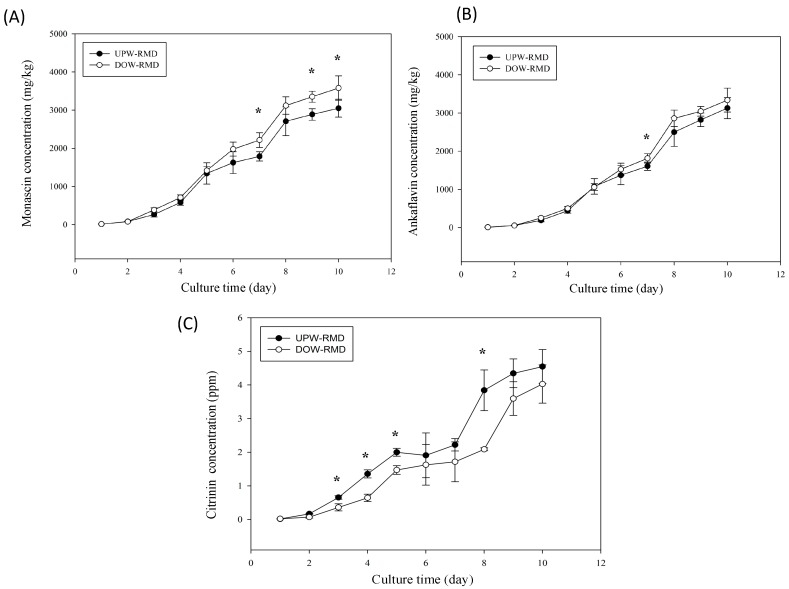
Time curve of the monascin (**A**); ankaflavin (**B**); and citrinin (**C**) concentrations of UPW-RMD and DOW-RMD. RMD was fermented with UPW or DOW at 28 °C. The data are presented as the means ± SD (*n* = 3). * *p* < 0.05, as compared with control.

To understand the influences that DOW has during the biosynthesis of *Monascus*-fermented metabolites, this study examined the metabolite biosynthesis curve produced by the fermentation process. Results illustrated in Figure 1 show that during the initial stage of fermentation, the addition of DOW failed to substantially increase MS and AK biosynthesis. However, DOW significantly facilitated the biosynthesis of MS and AK during the final stage of fermentation, achieving significant differences as compared with UPW (*p* < 0.05). Regarding the formation of CT, DOW inhibited CT formation during the initial stage of fermentation. Moreover, researchers observed a significant reduction in CT during the middle and final stages of fermentation when compared with UPW-RMD (*p* < 0.05). Therefore, following fermentation, the DOW-RMD demonstrated significantly increased MS and AK production, and reduced CT level.

### 2.2. Effect of the Individual Metal Waters on the Production of Monascus-Fermented Pigments

**Figure 2:**
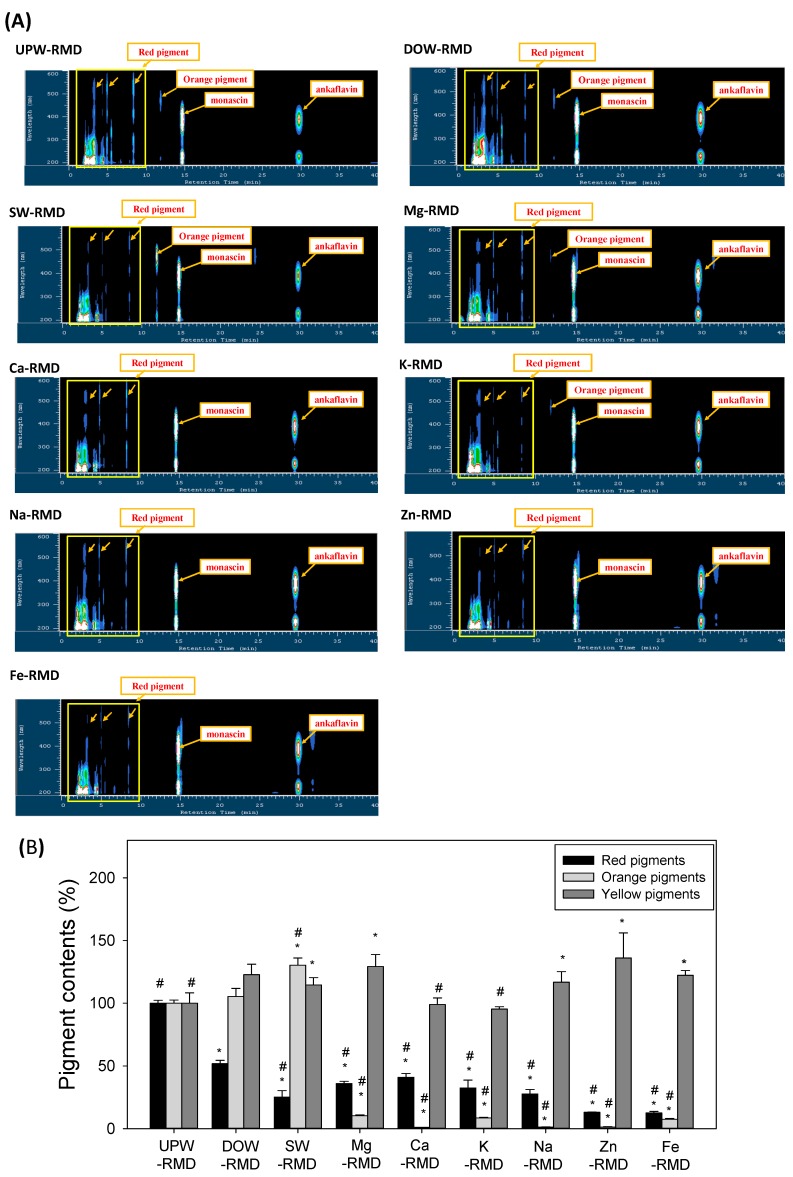
HPLC-PDA chromatograms (**A**) and quantification contents (**B**) of pigments of red mold dioscorea fermented with various metals in water. The data are presented as the means ± SD (*n* = 3). * *p* < 0.05, as compared with UPW-RMD group; ^#^ *p* < 0.05, as compared with DOW-RMD group.

To understand the influence that DOW has on the secondary metabolite biosynthesis of RMD, this study compared HPLC-PDA detection profiles of RMD cultured with various types of water (Figure 2A). The yellow pigment (MS and AK) contents presented two maximum absorption wavelengths at 230 and 400 nm, the orange pigment content presented a maximum absorption wavelength at 230 and 450 nm, and the red pigment content presented maximum absorption wavelengths at 300, 450, and 550 nm [20]. In addition, the retention times of peak for the yellow pigments (MS and AK) were 14.8 and 29.7 min, respectively. Figure 2B also show significantly reduced red pigment content in the DOW-RMD (*p* < 0.05 *vs.* UPW-RMD). However, the DOW-RMD presented higher yellow pigment contents than the UPW-RMD (*p* < 0.05). The SW was prepared based on the Mg, Ca, K, Na, Zn, and Fe concentrations in DOW to fabricate SW-RMD. However, the SW-RMD presented different pigment content than the DOW-RMD. The SW presented substantially less red and yellow pigment, but more orange pigment than the DOW-RMD (*p* < 0.05). These results suggest that the reason for the increase in MS and AK and the reduction in CT primarily originate from the metals composition. RMD was also fermented by using Mg, Ca, K, Na, Zn, or Fe water as the culture water. In Figure 2B, all the individual metal waters reduced red pigment biosynthesis (*p* < 0.05 *vs.* UPW-RMD), as well as inhibiting the biosynthesis of orange pigments (*p* < 0.05 *vs.* UPW-RMD). However, the Mg-RMD, Na-RMD, Zn-RMD, and Fe-RMD had significantly higher yellow pigment (MS and AK) contents than UPW-RMD (*p* < 0.05).

### 2.3. Effect of the Individual Metal Waters on the Production of Monascus-Fermented Metabolites

These results show that the metals contained in DOW influence the amount of biosynthesized MS, AK, and CT. To understand which metal in DOW increases MS and AK and reduces CT, the concentration of each metal water was prepared according to its concentration in DOW. This study also prepared each metal at 0.2, 1, and 5 times the original concentration to examine the changes in MS, AK, and CT levels of RMD.

**Figure 3:**
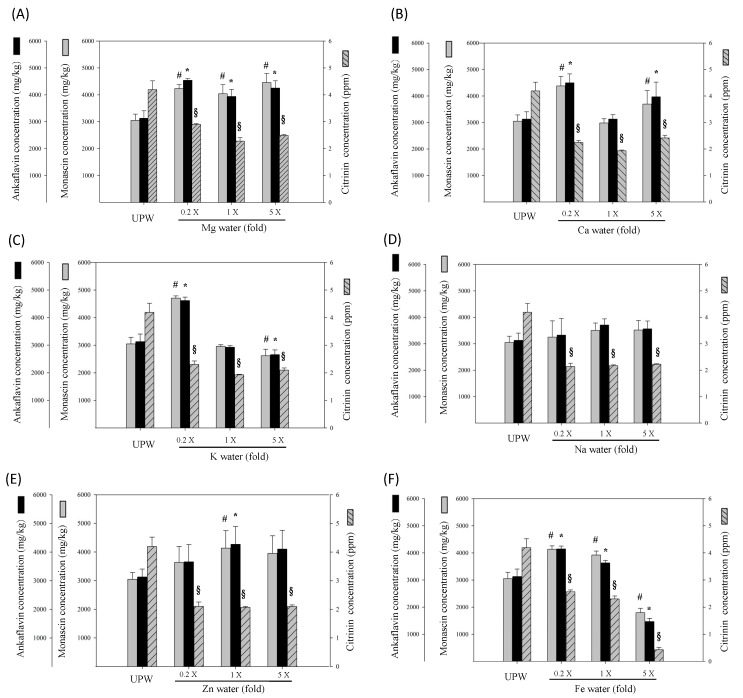
Effect of various metal water concentrations on the content of monascin, ankaflavin, and citrinin in the RMD. The metal concentration in the water was equal to that in deep ocean water. Concentrations are presented as the means ± SD (*n* = 3); ^#^ *p* < 0.05, as compared with control of monascin; * *p* < 0.05, as compared with control of ankaflavin; and ^§^ *p* *<* 0.05, as compared with control of citrinin.

The results illustrated in Figure 3A show that the Mg water significantly increased MS and AK production, gradually slowing as concentrations increased. For CT level, the addition of Mg water significantly reduced the CT level of RMD, achieving a dose effect (*p* < 0.05). This verified that the addition of Mg effectively increased MS and AK and reduced CT. In Figure 3B, culturing *Monascus* with the Ca water significantly increased MS and AK, with 0.2 times the original concentration (1.004 mg/L) presenting the highest production (*p* < 0.05 *vs.* UPW). However, formation gradually slowed as Ca water concentration increased. CT levels significantly decreased with the concentration of Ca water, particularly at the original concentration (5.02 mg/L) (*p* < 0.05 *vs.* UPW). However, five times the original concentration of Ca (25.1 mg/L) failed to further reduce CT. This verified that the addition of Ca effectively increased MS and AK and reduced CT. The influence of K water was shown in Figure 3C. The addition of K water at 0.2 times the original concentration (0.044 mg/L) significantly increased MS and AK productions. However, increasing K water concentration reduced MS and AK production and significantly reduced the CT level (*p* < 0.05 *vs.* UPW). Figure 2D indicates that the addition of high concentration of Na water still failed to significantly increase MS and AK production, but a significantly reduced CT level was achieved by a low concentration of Na water (*p* < 0.05 *vs.* UPW). In Figure 3E, the supplementation of Zn water significantly increased MS and AK production, and significantly reduced the CT level (*p* < 0.05 *vs.* UPW). Figure 3F indicates that RMD cultured in Fe water increased MS and AK productions, gradually slowing as concentrations increased. CT level reduced as Fe water concentration increased.

### 2.4. Proliferation and Differentiation of Preadipocytes

The abovementioned results show that the biosynthesis of *Monascus*-fermented metabolites varied with changes in the mineral composition of DOW, and that MS and AK production increased with the addition of certain minerals. Previous studies have indicated that MS and AK demonstrate anti-adipogenesis effects [19]. To understand whether the anti-adipogenesis ability of RMD changes when cultivated with different mineral waters, this study treated 3T3-L1 preadipocytes with various ethanol extracts of RMD (RE) cultured using various individual mineral waters (Mg-water, Ca-water, Na-water, K-water, Zn-water, and Fe-water) and found that the anti-differentiation and anti-lipogenesis effect differed with the various mineral-RE cultured in different mineral contents.

**Figure 4:**
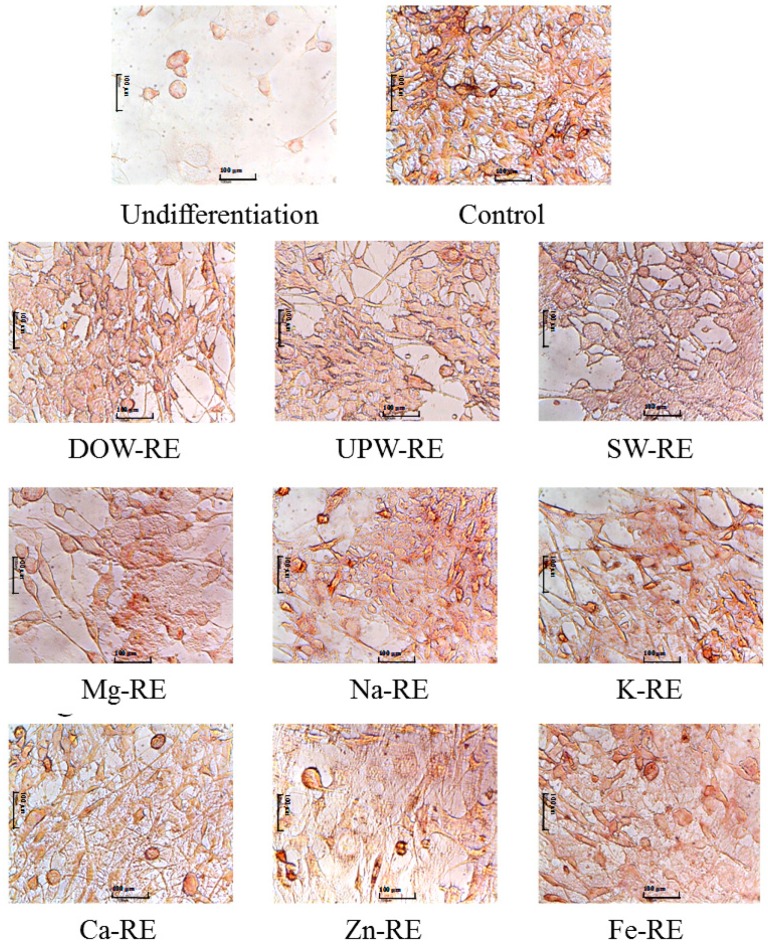
Effects of the ethanol extracts of RMD cultured with UPW, DOW, SW, and various metals water on 3T3-L1 preadipocyte differentiation. Preadipocytes were differentiated according to the method described in Materials and Methods. During differentiation the cells were treated with DOW-RE or UPW-RE. On day 8, the cells were fixed and stained with oil-red O.

Findings showed that DOW-RE, UPW-RE, SW-RE, and each metal-RE failed to significantly influence preadipocyte proliferation (data not shown). To evaluate the differentiation test, this study performed an oil-red O staining tested on the 3T3-L1 preadipocytes on Day 9 of induced differentiation. During differentiation, 3T3-L1 preadipocyte cells began to produce lipid droplets and to accumulate oil, causing the adipocyte to fatten and eventually differentiate into a mature adipocyte. Oil-red O combined with neutral fats and oils to form red lipid droplets. Results illustrated in Figure 4 show that Mg-RE, Zn-RE, and K-RE samples more effectively inhibited the formation of lipid droplets in the control and UPW-RE groups during the preadipocyte differentiation process.

### 2.5. Lipolysis and Lipogenesis of Mature Adipocyte

**Figure 5:**
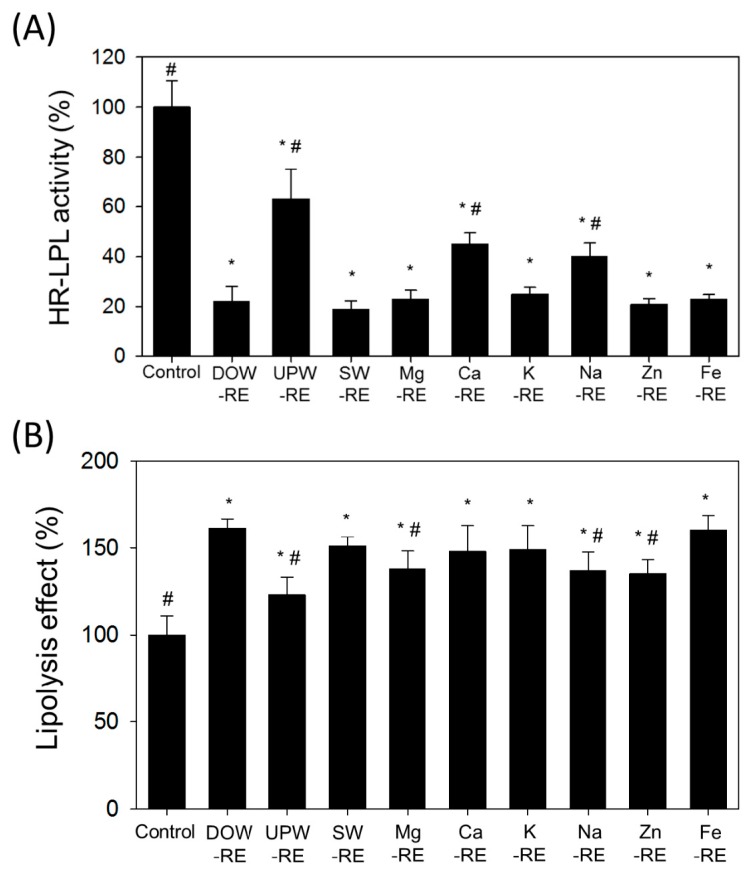
Effects of the ethanol extracts of RMD cultured with UPW, DOW, SW, and various metals in water on lipogenesis in mature 3T3-L1 adipocytes: (**A**) HR-LPL activity; (**B**) lipolysis effect. The data are presented as the means ± SD (*n* = 3). * *p* < 0.05, as compared with UPW-RMD group; ^#^ *p* < 0.05, as compared with DOW-RMD group.

LPL decomposes extracellular TG into free fatty acids and transports these acids into the cells. Cells then undergo lipogenesis and accumulate lipid droplets [22]. Results of Figure 5A showed that treating 3T3-L1 adipocytes with DOW-RE and SW-RE significantly reduced HR-LPL activity by 40% when compared with those treated with UPW-RE (*p* < 0.05). In addition, the various metal-RE also had significantly reduced HR-LPL activity. The Mg-RE, Na-RE, K-RE, Ca-RE, Zn-RE, and Fe–RE reduced HR-LPL activity by 25%, 48.4%, 27%, 41.5%, 21.5%, and 24.3%, respectively (*p* < 0.05), suggesting that RMD cultivated with water including specific metals contained in DOW or SW increases the effectiveness in reducing HR-LPL activity, and has a potent effect similar to DOW-RE and SW-RE.

Free fatty acid and glycerol are products of triglyceride (TG) hydrolysis. Free fatty acids are released extracellularly or undergo β-oxidation intracellularly to serve as energy or as material for subsequent synthesis of TG. Therefore, increasing lipolysis ability facilitates the reduction of the accumulation of lipid droplets [22]. Results of Figure 5B for treating adipocytes with DOW-RE, UPW-RE, SW-RE, and various metal-RE samples showed that Mg-RE, Na-RE, K-RE, Ca-RE, Zn-RE, and Fe-Re demonstrated a higher lipolysis ability than UPW-RE (*p* < 0.05). The lipolysis ability of Fe-RE was similar to that of DOW-RE (*p* > 0.05).

## 3. Discussion

Previous studies have indicated that DOW effectively reduces CT biosynthesis and increases MS and AK biosynthesis, thereby enhancing the health functions of RMD [12,20,21]. To understand the influences that the major metals contained in DOW have on regulating metabolite production and various health functions, this study prepared six types of major metal water samples based on the concentrations of these individual metals contained in DOW. The various metal-waters were used to culture RMD, which were examined to determine the influences that the six major metals have on the production and anti-adipogenesis effect of *Monascus*-fermented metabolites.

The question of whether DOW can be artificially produced to replace SW is often raised in studies related to DOW and in related industries. The findings of the present study showed that the results of SW and DOW were different. CT level was inhibited with the addition of DOW and SW. AK production increased with DOW, but was inhibited with SW. SW was able to more effectively inhibit red pigment biosynthesis and increase orange pigment biosynthesis than DOW. Moreover, the concentrations of the six metals contained in SW were prepared based on the metal concentrations contained in DOW. The metal composition of SW effectively influenced the pigment metabolism and inhibited the CT level of RMD, but the effects and trends regulated by SW were still different from those regulated by DOW. These findings were consistent with those of previous studies [20], which suggested that SW is the default to replace DOW in the regulation of *Monascus*-fermented metabolite production. These differences may be because of the influence that metal ions and trace elements in DOW have on the metabolite biosynthesis of *Monascus*. Although the types of major metals used in the present study did not encompass all metal types contained in DOW, they were the metals with the highest concentration. This study used a mixture of these major metals to prepare the SW used to culture *Monascus* in the attempt to understand the influence that the major metals in DOW and SW have on regulating *Monascus*-produced metabolites. These findings can serve as a valuable reference for improving electric dialysis and water separation techniques based on DOW.

Findings obtained in this study verified that the major metals contained in DOW influence *Monascus*-produced CT level, whereas the addition of SW (*i.e.*, Mg, Ca, K, Na, Zn, and Fe) reduced the CT level. CT is a mycotoxin biosynthesized by *Monascus*, and *Monascus* is produced by microorganisms present in stress environments. Metals consequently facilitate the biosynthesis of *Monascus*, and reduce stress conditions that may reduce the level of CT. Another factor may be that metals regulate pigments’ biosynthesis pathways, which indirectly influence CT biosynthesis. CT is a polyketide derivative, which is a branch of the red pigment biosynthesis pathway [23,24]. Numerous studies have indicated that polyketide products, such as red pigments, increase in proportion to CT biosynthesis [23]. Based on the results, the Mg, Ca, K, Zn, Na, and Fe water samples significantly inhibited CT, red pigment, and orange biosynthesis (*p* < 0.05 *vs.* UPW). Moreover, the metal samples diverted to various productions of yellow pigments (MS and AK). Functional yellow pigment production can be increased by the supplementation of Mg, Na, Zn, and Fe water but not by the supplementation of K and Ca water. However, the SW resulted in different effects as compared with the individual metal waters. SW significantly increased the production of orange pigment even though SW consisted of the six metal waters, which gave the opposite result. The results suggest that the combination of the six individual metal waters may have a synergistic effect on the regulation of pigments biosynthesis.

The aforementioned discussion verified that different metals regulate different pigment biosynthesis pathways. Therefore, metal concentrations also influence the biosynthesis of functional metabolites—MS and AK. Low concentrations of Mg, Ca, K, Zn, and Fe water inhibit the CT level, as well as elevate MS and AK production. When increasing metal concentration to five times the original concentration, K and Fe water significantly inhibited MS and AK production (*p* < 0.05 *vs.* UPW). The remaining metals failed to further increase MS and AK production when increasing their concentrations. High metal concentrations may cause stress conditions, which reduce *Monascus* growth and metabolism and decrease the overall biosynthesis of metabolites. Findings further revealed that five times the original concentration of Fe water reduced the biosynthesis of red, orange, and yellow pigments. Therefore, this study inferred that the metals in water reduce red pigment biosynthesis and inhibit CT by regulating pigment biosynthesis and conversion, consequently enhancing the biosynthesis of functional yellow pigments such as MS and AK.

Previous studies have confirmed that RMD cultivated using DOW exhibits increased health functions, which include hypolipidemic, anti-atherosclerosis, and anti-obesity functions [12,20,21]. DOW effectively inhibits the performance of transcription factor C/EBPβ of adipocytes during differentiation, consequently reducing the mRNA expression of C/EBPα and PPARγ to achieve body fat-lowering effects [6]. These studies considered the improvement in health to be the result of the positive influence that DOW had on the MS and AK content of RMD, which improves RMD’s hypolipidemic and anti-obesity effects [13,21]. Previous studies have indicated that the anti-obesity effects of MS and AK typically derive from the inhibition of differentiation of preadipocytes and the stimulation of mature adipocyte lipolysis [19]. The DOW-RMD possessed DOW and higher concentrations of MS and AK. Therefore, previous studies have contended that DOW-RMD possesses superior anti-obesity effects [21]. However, the influence that the metal composition in DOW has on the anti-obesity effects of RMD is unclear.

Research findings further suggest that the DOW-RMD primarily facilitated the lipolysis effect of mature adipocytes, and inhibited subsequent lipogenesis. In addition, the DOW-RMD more effectively inhibited the proliferation and differentiation of preadipocytes. However, the SW-RE presented similar performances in facilitating lipolysis and inhibiting lipogenesis when compared with the DOW-RE, suggesting that the metal composition in SW may be the important factor of DOW for promoting the anti-adipogenesis effect of RMD. The six RE samples prepared with six types of metals significantly increased lipolysis, outperforming UPW-RE. Regarding the regulation of lipogenesis, the inhibition effects of the RE samples of DOW and SW outperformed that of UPW-RE known as an anti-adipogenesis sample. This study inferred that the more potent anti-lipogenesis effect of SW-RE originated from the metal content, specifically Mg, K, Zn, and Fe in SW, and therefore we deduced that this metal content was the reason the DOW-RE could effectively inhibit lipogenesis. However, whether these effects were derived only from the increase in MS and AK remains to be determined. The Mg-RE, Zn-RE, and Fe-RE samples presented higher MS and AK biosynthesis than the UPW-RE samples, which enhanced their lipogenesis inhibition abilities. However, previous studies have indicated that SW has shown no lipogenesis inhibition ability, but that DOW has exhibited this ability [21]. Therefore, the lipogenesis inhibition ability demonstrated by the SW-RE samples may not have originated only from the six types of metals contained in SW. Findings indicated that although the K-RE failed to significantly increase MS and AK biosynthesis, these samples demonstrated superior lipogenesis inhibition. The researchers inferred that the addition of K water may have caused *Monascus* to develop other functional components that inhibited mature 3T3-L1 adipocyte HR-LPL activity and realized lipogenesis inhibition.

## 4. Experimental Section

### 4.1. Chemicals

LC grade acetonitrile, chloroform, methanol, and dimethyl sulfoxide (DMSO) were purchased from Merck Co. (Darmstadat, Germany). Tryptone, yeast extract, peptone, malt extract, potato dextrose agar (PDA), and Bacto-agar were purchased from Difco Co (Detroit, MI, USA). Dulbecco’s modified Eagle’s medium and fetal bovine serum were purchased from Invitrogen Life Technologies (Carlsbad, CA, USA). Dexamethasone, isobutylmethylxanthine, insulin, oil-red O, heparin, *p*-nitrophenyl butyrate, and the standard solutions of Mg, Na, K, Ca, Zn, and Fe were purchased from Sigma Chemical Co (St. Louis, MO, USA). Trypan blue stain was purchased from Gibco BRL Life Technologies Inc. (Gaithersburg, MD, USA).

### 4.2. The Source of DOW and the Preparation of Synthetic Water (SW) and the Various Metal Waters

The concentrated DOW provided from the Eastern Taiwan Deep Sea Water Innovation and Research Center (Taitung, Taiwan) was pumped from a depth of 670 m in the Pacific Ocean near Eastern Taiwan and processed though electrodeionization and vacuum concentration. The concentrations of the trace elements and minerals in DOW including Al, Cu, Zn, As, Ba, Cd, Cr, Pb, Hg, Se, Ag, Ca, Mg, K, Na, Sb, Tl, Be, fluoride, nitrate, sulfate, chloramines, and chlorine have been measured and published in our previous study [15]. SW was prepared by mixing the main ions of DOW including 20.65 mg/L Mg, 5.02 mg/L Ca, 7.71 mg/L Na, 0.22 mg/L K, 0.0062 mg/L Fe, and 0.019 mg/L Zn ion with equal concentrations to those in DOW. The metal water (Mg, Ca, K, Na, Zn, and Fe water) was prepared by the dilution of metal standard solution according to that metal’s concentration in the DOW.

### 4.3. Preparation of UPW-RMD, DOW-RMD, SW-Cultured RMD (SW-RMD), and Individual Metal-RMD

*Monascus purpureus* NTU 568 (DSM 28072; International depositary authority: Leibniz Institute DSMZ-German Collection of Microorganisms and Cell Cultures) fermented product has been proven to have a potent hypolipidemic effect in our previous study [17,25]. The culture strain was maintained on PDA slant at 10 °C and transferred monthly. The dioscorea root (*Dioscorea batatas* Dence), purchased from a local supermarket in Taiwan, was used to produce RMD using the method of solid-state culture. UPW, DOW, SW, and various metal waters were used in the production of UPW-RMD, DOW-RMD, SW-RMD, and metal-RMD. The individual metal-RMDs, including Mg-RMD, Ca-RMD, Na-RMD, K-RMD, Zn-RMD, and Fe-RMD, were fermented using Mg water, Ca water, Na water K water, Zn water, and Fe water, respectively. However, the concentration of each metal in water was equal to that in DOW. After fermentation, crushed and dried RMD powder was used for the experiments [20,26].

### 4.4. Determination of Monascin, Ankaflavin, and Citrinin Concentration of RMD

RMD (1 g) was extracted with 10 mL of 75% ethanol at 60 °C for 30 min. The extracts (10%, *w*/*v*) were further filtered with a 0.45-μm pore size filter and analyzed by HPLC (Model L-2130, Hitachi Co., Tokyo, Japan). HPLC was performed according to the method described previously in triplicate [27]. Monascin and ankaflavin were detected using a photodiode array detector (PDA) (Model L-2455 DAD, Hitachi Co.) set at 231 nm. For citrinin analysis, the fluorescence detector (Model L-2485 FL Detector, Hitachi Co.) was set with an excitation wavelength of 330 nm and an emission wavelength of 500 nm.

### 4.5. Determination of the Levels of Red, Yellow, and Orange Pigments of RMD

The content of yellow pigments was the sum of the contents of monascin and ankaflavin. Red pigments have specific high absorption wavelengths at 300 nm, 400 nm, and 530 nm. Orange pigments have specific high absorption wavelengths at 230 nm and 450 nm. The total integral values of the specific peaks of red pigments or orange pigments were detected using HPLC-PDA [20].

### 4.6. Cell Culture

3T3-L1 preadipocytes purchased from the Bioresource Collection and Research Center (Hsinchu, Taiwan) were cultured in basal medium (Dulbecco’s modified Eagle’s medium containing 10% fetal bovine serum) at 37 °C in 5% CO_2_. To induce differentiation, two-day postconfluent 3T3-L1 preadipocytes (day 0) were stimulated for 48 h with 0.5 mM isobutylmethylxanthine, 1 mM dexamethasone, and 10 mg/mL insulin (MDI) added to the basal medium. On day 2, the MDI medium was replaced with a basal medium containing insulin only. On day 4 and thereafter, the cells were cultured in a basal medium, which was changed every two days until the cells were analyzed.

DOW-RE, SW-RE, UPW-RE, and various metals-RE (Mg-RE, Ca-RE, K-RE, Na-RE, Fe-RE, and Zn-RE) were prepared using the extraction of DOW-RMD, SW-RMD, UPW-RMD, and various metals-RMD (Mg-RMD, Ca-RMD, K-RMD, Na-RMD, Fe-RMD, and Zn-RMD) with 10-fold volume of 95% ethanol at 37 °C for 24 h, respectively. DOW-RE, SW-RE, UPW-RE, and various metals-RE were diluted to various concentrations with DMEM medium, and further used as the treatment medium in the cell experiments. The vehicle control was 0.3% ethanol in culture medium, which was equal to the ethanol concentration in all RMD extract treatments.

### 4.7. Oil-Red O Staining

Differentiated 3T3-L1 cells on day 8 were fixed with 10% formaldehyde and then stained with oil-red O. Pictures were taken using a microscope (ECLIPSE TS100; Nikon Co., Tokyo, Japan) [28].

### 4.8. Lipolysis Assay

The fully differentiated 3T3-L1 adipocytes (days 8–12 after differentiation induction) were treated with test substances in Krebs Ringer bicarbonate (KRB) buffer (20 mM NaCl, 4.7 mM KCl, 2.2 mM CaCl_2_, 1.2 mM MgSO_4_·7H_2_O, 1.2 mM KH_2_PO_4_, 25 mM NaHCO_3_ and 2% BSA; pH 7.4) for 24 h [29]. Glycerol was determined enzymatically from the supernatant by using a kit (Randox Laboratories Ltd., Antrim, UK).

### 4.9. Heparin-Releasable Lipoprotein Lipase (HR-LPL) Activity Assay

After incubation of the 3T3-L1 mature adipocytes with the experimental medium for 24 h, the medium was discarded. The cells were rinsed with KRB buffer and then cultured in heparin-KRB (10 U/mL heparin) at 37 °C for 1 h. The conditioned heparin-KRB was collected from each well for the assay of HR-LPL activity. LPL activity was measured according to the previous study on the basis of its esterase property using *p*-nitrophenyl butyrate as a substrate [28].

The TG hydrolase activity of LPL with synthetic TG substrates is inhibited by molar sodium chloride, and this property has been used to distinguish LPL activity from the activities of other lipases. Thus, HR-LPL activity was calculated from the productivity of *p*-nitrophenol using the following equation [28]:

*C (*μ*M) = (A_400_ (0.15 M NaCl) − A_400_ (1 M NaCl))/0.012*

where A_400_ (0.15 M NaCl) and A_400_ (1 M NaCl) were the absorbances of released *p*-nitrophenol at 400 nm in 0.15 M and in 1 M NaCl assay buffer, respectively, and 0.012 is the micromolar extinction coefficient of *p*-nitrophenol.

### 4.10. Statistics

Data are expressed as means ± standard deviation. Analysis of variance by Duncan’s test and Pearson’s product-moment correlation coefficient test were determined using SPSS version 10.0 software (SPSS Institute, Inc., Chicago, IL, USA). Differences with *p* < 0.05 were considered statistically significant.

## 5. Conclusions

Previous studies have confirmed that DOW positively influences pigments such as anti-adipogenesis compounds, MS, and AK, as well as the CT and health functions of *Monascus.* The present study verified that these positive changes originate from the metals contained in DOW for the first time. The addition of Mg, Ca, Zn, and Fe water increased MS and AK production and inhibited the CT level. The combination of individual metals may express a synergistic effect, which may be a result of the inhibition of red pigment biosynthesis, stimulating pigments to follow yellow pigment biosynthesis pathways. The metabolic conversion caused by such changes may influence health functions. When testing the differentiation and lipogenesis of adipocytes, the Mg-RE, K-RE, Zn-RE, and Fe-RE samples demonstrated the highest lipogenesis inhibition. Therefore, this study expanded the argument proposed in previous studies that DOW-RMD significantly enhances anti-obesity effects by inferring that these effects may originate from the metals (*i.e.*, Mg, Ca, Zn, Fe) contained in DOW. These metals stimulated MS and AK production, which increased the generation of fermented products to inhibit mature adipocyte lipogenesis. Moreover, the addition of K failed to significantly increase functional components, but demonstrated significant lipogenesis inhibition. These results suggest that other functional components of *Monascus* that inhibit lipogenesis may have also increased. Although the SW employed in the present study failed to replace DOW in becoming the optimal type of water for fermenting RMD, the results highlighted the significance of DOW and provided a clearer understanding of the roles of the major metals contained in it.
